# Goteo.org civic crowdfunding and match-funding data connecting Sustainable Development Goals

**DOI:** 10.1038/s41597-020-0472-0

**Published:** 2020-04-29

**Authors:** Mayo Fuster Morell, Enric Senabre Hidalgo, Enrique Rodríguez

**Affiliations:** 1 Universitat Oberta de Catalunya, Barcelona, Spain; 2 Austrian Academy of Sciences, Vienna, Austria

**Keywords:** Interdisciplinary studies, Sociology

## Abstract

The United Nations’ Sustainable Development Goals (SDGs) highlight priority areas for global sustainable development, such as reducing inequalities and protecting the environment. Digital platforms, such as Goteo.org, facilitate financial support from individuals for SDG-related initiatives through crowdfunding and match-funding campaigns. Match-funding is a type of crowdfunding, where individual donations are matched or multiplied by public and private organizations. There remains a lack of open data, however, to study the effectiveness of match-funding as a way to finance these civic initiatives. The Goteo.org platform’s approach to data transparency and open source principles have allowed these data to be collected, and here we present a dataset for 487 civic crowdfunding campaigns. This dataset presents a unique opportunity to compare the behaviour of different crowdfunding modalities in parallel with the SDGs.

## Abstract (summary)

**Table:** 

Measurement(s)	crowdfunding campaigns • Donation
Technology Type(s)	Goteo digital platform • digital curation

Machine-accessible metadata file describing the reported data: 10.6084/m9.figshare.12053847

## Background & Summary

Civic technologies^1^ can facilitate the analysis of tools and digital platforms from different perspectives for purposes such as social justice, socio-economic development, improving the functioning of government or facilitating communication between citizens^2–4^. The study of crowdfunding is one of the most significant in this area^5^. Crowdfunding allows creators and entrepreneurs to receive individual donations to finance their projects through specific online campaigns. In exchange for predetermined donations from users, campaign promoters offer a series of rewards or gratitude returns. Since the proliferation of crowdfunding platforms in the early 2000s, various modalities and operating formulas have evolved, as well as research on the phenomenon. However, analyses specifically relating to civic crowdfunding are relatively scarce and most empirical studies focus on either addressing technological mechanisms “per se”^6^, their effectiveness as a generic funding channel (regardless of themes)^7^, or on the disruptive impact of crowdfunding on the cultural sphere^8^.

In recent studies, authors have examined crowdfunding’s potential from the perspective of public administration, considering hybrid models of crowdfunding and public funding^9–11^. Brent and Lorah^12^ investigate how policymakers can acquire knowledge about society’s preferences through crowdfunding, while Hong and Ryu^13^ demonstrate how public entities can contribute to improving the efficiency of crowdfunding campaigns. In that sense, match-funding is one modality that has emerged in recent years. It is crowdfunding “as usual,” but with individual donations complemented by funds from collaborating organizations. These additional funds increase the probability of the project’s success and the average amount donated^14^. Usually, the objectives for public administration align around a common good, while for private entities, they relate to corporate social responsibility^15^. Although the more common-place match-funding model only allows organizations to contribute complementary funds at the end of an established campaign period (depending on whether the initial project funding objectives are achieved), there are more sophisticated variations where each citizen’s contribution is instantly multiplied by the collaborating institution. This mode of “dynamic” match-funding results in a more transparent process, where each contribution is visualised in real-time through the platform interface^16^.

When considering the impact of tools such as civic crowdfunding, it is important to use transversal approaches that connect to social needs and problems relating to global challenges^17,18^. The United Nations’ (UN) Sustainable Development Goals (SDGs), an initiative promoting the implementation of an ambitious development agenda by 2030 addressing issues such as climate change, economic inequality and sustainable consumption, represents international indicators starting to be adopted by all types of international institutions^19^. Given that the SDGs were only introduced in 2015, analyses of their implementation and impact in local contexts are still scarce^20^, as its connection with civic mobilisation beyond institutional actions. However, the need and urgency to prioritize them in international research and sustainable development efforts are widely accepted^21^.

Although some recent studies quantitatively address the progress and systemic interrelation of each of the 17 SDGs^22^, and even their possible connection with crowdfunding^23^, our approach makes the first contribution to both fronts from an open data perspective, adding to an emerging trend in the study of the impact of civic technologies^24^. The dataset examines civic crowdfunding, match-funding and the SDGs from two dimensions: (1) the efficiency and behaviour through usual crowdfunding models in contrast with purely match-funding mechanisms; and, (2) their connection with the transversal priorities of the SDGs.

It is important to emphasise for other studies to which this dataset can contribute regarding the financing of the SDGs (with a total volume reflecting 3,497,502 euros among the 487 Goteo campaigns included, from 55,419 donations), that whilst it can serve as an indicator for different approaches and datasets, in a macro context, it represents a limited scope when compared to general figures from international organizations and public and private financing; some experts have estimated that to meet the SDGs globally by 2030 requires annual funding of trillions of euros^25^.

## Methods

Regarding the first dimension, distinguishing between modalities of civic crowdfunding, this dataset covers results from 487 crowdfunding campaigns of different types on the Goteo digital platform (https://www.goteo.org/), between February 2017 and May 2019. Goteo represents a unique approach to data transparency as one of the few open source crowdfunding platforms in the world^16^, allowing full public scrutiny of its main funding dynamics, campaigns and backer behaviours. The dataset includes the typology of campaigns, differentiating between those following the usual crowdfunding mechanism (392) and those with match-funding models (95) which have been implemented by the platform in recent years for various pilot projects. Among the latter, the dataset distinguishes between campaigns that have applied match-funding which supplements the donations received at the end of the campaign period (the usual format in crowdfunding platforms experimenting with this model), and those which have dynamically multiplied individual donations from users in real time (through an “ad hoc” formula developed by Goteo). Likewise, through the main dataset provided, each campaign is accompanied by descriptive data (title, subtitle, description of objectives, motivation, social commitment, etc.) and also data on publication date, original language and URL, as well as the amount of money requested, obtained, and other relevant funding statistics.

Regarding the dimension of civic projects funded by civil society concerning each SDG theme, this dataset is innovative, presenting a detailed coding based on a double validation process. Firstly, automatic coding according to “social commitments” as defined by users as campaign promoters, followed by a phase of manual coding in which we have reviewed and refined the specific relationship with one or more of the SDGs in each of the 487 campaigns. This has been based on the presentations and textual contents of each one. Adding more possible elements of discussion to the still emergent literature on SDGs and economic impact^26^, this dataset allows for establishing a series of relationships and observations among a corpus of data on civic crowdfunding campaigns of different modalities according to their detailed classification regarding SDGs.

As such, each campaign is accompanied by a set of additional data describing its connection with the 17 SDGs, allowing for comparative analysis beyond crowdfunding modalities and relating to the themes of each goal:

- Goal 1: End poverty, in all its forms everywhere.
- Goal 2: End hunger, achieve food security, improve nutrition and promote sustainable agriculture.
- Goal 3: Ensure healthy lives and promote well-being for all.
- Goal 4: Ensure inclusive and equitable quality education and promote lifelong learning opportunities for all.
- Goal 5: Achieve gender equality and empower all women and girls.
- Goal 6: Ensure availability and sustainable management of water and sanitation for all.
- Goal 7: Ensure access to affordable, reliable, sustainable and modern energy for all.
- Goal 8: Promote sustained, inclusive and sustainable economic growth, full and productive employment and decent work for all.
- Goal 9: Build resilient infrastructure, promote inclusive and sustainable industrialization and foster innovation.
- Goal 10: Reduce inequality within and among countries.
- Goal 11: Make cities and human settlements inclusive, safe, resilient and sustainable.
- Goal 12: Ensure sustainable consumption and production patterns.
- Goal 13: Take urgent action to combat climate change and its impacts.
- Goal 14: Conserve and sustainably use the oceans, seas and marine resources for sustainable development.
- Goal 15: Protect, restore and promote sustainable use of terrestrial ecosystems, sustainably manage forests, combat desertification, halt and reverse land degradation, halt biodiversity loss.
- Goal 16: Promote peaceful and inclusive societies for sustainable development, provide access to justice for all and build effective, accountable and inclusive institutions at all levels.
- Goal 17: Strengthen the means of implementation and revitalize the global partnership for sustainable development.

The Goteo platform does not explicitly facilitate the connection of crowdfunding campaigns with the SDGs, so potential donors (also called ‘backers’) are guided exclusively by how each promoter explains the specific theme and civic commitments of their projects. However, since its inception, the platform has had a system of classification by themes that allows access to campaigns which (as described below) has evolved to allow an automatic initial reclassification of such themes around the SDGs, before internal review and manual coding.

### Data collection and coding process

Goteo, besides its focus on civic crowdfunding campaigns, is characterised by being open source and facilitating a series of open data through an API. From 1,383 projects published between the time of writing and the start of operations on the platform at the end of 2011 (a total volume of more than 117,000 backers) data of active campaigns during 39 months were chosen for this dataset. The cut-off date for the dataset (instead of covering all the campaigns since the start of the platform) was decided based on a new thematic classification for Goteo campaigns introduced by the promoters of the platform (the non-profit, Goteo Foundation). Since February 2017 projects can be classified according to an impact model called “social commitment”, differing from the original ones of:

- Solidary
- Free & libre software
- Creating employment
- Independent journalism
- Education
- Creating culture
- Ecologic
- Open data
- Strengthen democratic values
- Citizen participation
- Gender
- Health & care

The data collection process, once the initial corpus of 487 campaigns was identified, involved associating a series of additional fields linked to each of the SDGs, to the first version of the dataset (regarding descriptive and performance data). These fields came from the automatic assignment of a positive or negative relationship of campaigns (values of 0 or 1) with each of the 17 SDGs, according to a matrix of analogies between the social commitments reflected in the list above - a codification agreed upon previously by the promoters of the platform.

Subsequently, two researchers manually reviewed the codification of all the data linked to the SDGs for each of the 487 campaigns, refining the automatic classification and in most cases limiting the relationship with SDGs to the three most relevant categories. The validation of this second coding was addressed by two specific meetings among researchers and members of Goteo staff, to check and discuss the results of a preliminary pilot coding of 50 campaigns. The variation to the initial automatic classification was 98%, as a percentage of campaigns to which a pre-assigned SDG category was added or removed. This resulted in a significant improvement of the categories linked to the SDG in the dataset after the manual review of the initial automatic coding, which was also discussed and validated in a final meeting between researchers and the Goteo platform promoters.

**Fig. 1:**
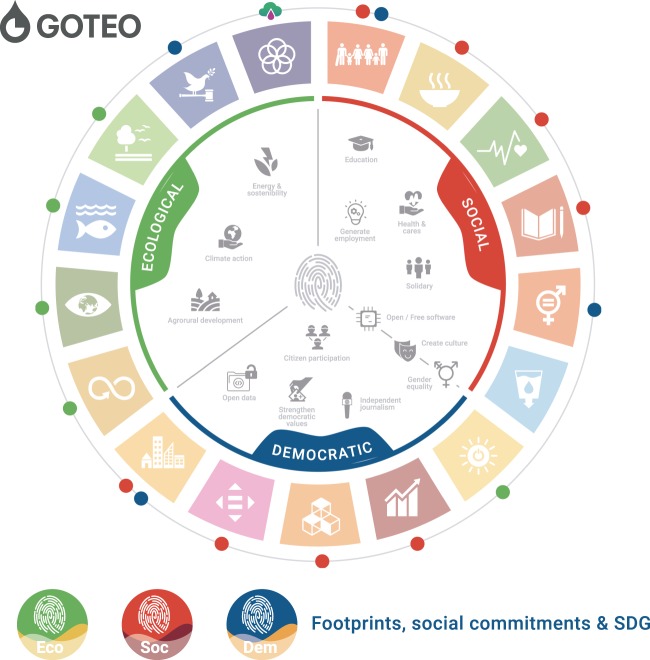
Classification of the SDGs in Goteo according to social commitment classification and the ecological, social and democratic footprints.

The dataset also provides a series of additional fields that come from a clustering of the different SDGs into three categories, called “footprints”. These allow for the visualisation of additional relationships according to the data of the automatic coding of SDGs: social, ecological and democratic (Fig. 1).

Finally, to facilitate the use of the dataset by third parties beyond campaign behaviour statistics, an English translation of the descriptive fields is used for all the initiatives from their original language (e.g. Spanish, Catalan, Galician, Basque), allowing textual content analysis to be performed (with a volume of more than 35,000 words in total).

Regarding the provenance, use and license of the data of this work, during the sign-up process, new users of the Goteo platform are informed about terms, conditions and privacy regarding data. Specifically: “in relation to how some activity data can be reproduced, publicly communicated, transformed or freely extracted in part or in whole, by anyone, in any format, with no restriction of time or territory, for any further legitimate use, but containing no personal data from individuals, in compliance with General Data Protection Regulation (EU) 2016/679 (GDPR)”. In this regard, our study is developed on publicly available open data sources, accessible via the Goteo API (http://developers.goteo.org/) as well as the contents of the platform itself at https://en.goteo.org/, and shared under the same conditions of the Creative Commons 3.0 BY-SA license, which is in force on the platform. In relation to public availability of the data used for the project, our study has not required the approval or review of an institutional ethics board. Regarding the availability of the unprocessed data (as in the case of untranslated versions of specific fields), the same sources indicated here can apply for direct access to it.

## Data Records

The following list describes the different values of the fields in the dataset^27^. Again, to increase the use of the dataset by diverse users in various locations and organizations, the original content has been translated to English, except where indicated.

### Goteo dataset coded

This is the main dataset covering the descriptors of 487 Goteo campaigns, after the automatic coding and manual coding processes explained above. The values relate to campaign descriptors originally user-generated by campaign organizers and project leaders, providing content via the Goteo registration form.

- **Project identifier**

- PROJECT ID - Project identification based on keywords (in different languages, depending on the original version).
- NAME - Project name, as reflected on the Goteo platform (in its original language, not translated to English).
- SUBTITLE - Text content with the full subtitle of the campaign, as reflected on the website (translated to English).
- URL - Active URL of the campaign page.

- **Crowdfunding modality**

- FUNDING TYPE - Regular crowdfunding behaviour in the campaign is reflected by the value NO MATCHFUNDING + NO MATCHER, while the other two values indicate dynamic match-funding or funds added by a matcher institution after the minimum goal was reached.
- MATCHFUNDING CALL - Values in this column refer to the match-funding calls to which they relate. If the value is NO it means that they behave like regular crowdfunding campaigns (with no automatic matching of funds).
- MATCHER - If values in this column are ahoracomparte it means that the campaign has received additional matching funds at the end of the funding period, if the minimum amount defined was reached.
- MATCHFUNDING_D - If the campaign is assigned to a specific match-funding call, the value is YES.
- MATCHER_D - With the value ‘yes’ if the campaign has a special assignment of a “matcher” institution, funds are added if the minimum funding goal is reached.

- **Campaign descriptors**

- PUBLISHED - Indicates the date on which the campaign is launched.
- FINISHED - Indicates the date on which the campaign finished.
- LANG - Indicates the original language in which the campaign was created: ca (Catalan); en (English); es (Spanish); eu (Eskara); fr (French); gl (Galego); sv (Swedish).
- DESCRIPTION - Text content with the full main description of the campaign and the objective of the project or initiative (translated to English).
- GOAL - Text content with a short description of the main goals of the campaign.
- MOTIVATION - Text content with a short description of the motivation of the project or initiative behind the campaign.
- ABOUT - Text content with a short bio or explanation about the people or organizations promoting the campaign.
- LEGAL TYPE - Information provided by project promoters about their legal status (Civil organization, Cooperative, NGO, Freelance, Private company, Individual, Other).
- LOCATION - Text content with the location of the project (city, region and/or country) as provided by campaign promoters, in the original language.
- SOCIAL COMMITMENT - Text content with a short description of the social commitment derived from the project or the objective of the campaign initiative.
- SC ID - Unique numeric identifier related to the social commitment of the campaign, as selected by the users when describing it via the Goteo form.

- **Sustainable Development Goals associated to each campaign**

- sdg_1 - End poverty in all its forms everywhere.
- sdg_2 - End hunger, achieve food security and improved nutrition and promote sustainable agriculture.
- sdg_3 - Ensure healthy lives and promote well-being for all at all ages.
- sdg_4 - Ensure inclusive and equitable quality education and promote lifelong learning opportunities for all.
- sdg_5 - Achieve gender equality and empower all women and girls.
- sdg_6 - Ensure availability and sustainable management of water and sanitation for all.
- sdg_7 - Ensure access to affordable, reliable, sustainable and modern energy for all.
- sdg_8 - Promote sustained, inclusive and sustainable economic growth, full and productive employment and decent work for all.
- sdg_9 - Build resilient infrastructure, promote inclusive and sustainable industrialization and foster innovation.
- sdg_10 - Reduce inequality within and among countries.
- sdg_11 - Make cities and human settlements inclusive, safe, resilient and sustainable.
- sdg_12 - Ensure sustainable consumption and production patterns.
- sdg_13 - Take urgent action to combat climate change and its impacts.
- sdg_14 - Conserve and sustainably use the oceans, seas and marine resources for sustainable development.
- sdg_15 - Protect, restore and promote sustainable use of terrestrial ecosystems, sustainably manage forests, combat desertification, halt and reverse land degradation and halt biodiversity loss.
- sdg_16 - Promote peaceful and inclusive societies for sustainable development, provide access to justice for all and build effective, accountable and inclusive institutions at all levels.
- sdg_17 - Strengthen the means of implementation and revitalize the global partnership for sustainable development.

- **Footprint codification**

- FP ID 1 - Ecological footprint: automatic assignment of values depending on the social commitment category of each campaign.
- FP ID 2 - Social footprint: automatic assignment of values depending on the social commitment category of each campaign.
- FP ID 3- Democratic footprint: automatic assignment of values depending on the social commitment category of each campaign.

- **Campaign performance**

- STATUS - Indicates if the campaign was successfully funded (irrespective of its modality) with “funded” or if not with “unsuccessful”.
- AMOUNT - Indicates the final amount of money received by the campaign, if successful (in euros).
- BACKERS- Indicates the total number of users who donated to the campaign (including matching institutions).
- NEW BACKERS - Indicates the number of new Goteo users donating to a campaign (that is, new backer accounts created during the campaign duration which donate to it).
- MULTIBACKERS - Indicates existing Goteo users (backers from other projects) that donate to a given campaign.
- MINCOST - Indicates the minimum funding goal indicated by the campaign promoters (under an “all or nothing” scheme).
- MAXCOST - indicates the maximum funding needed for the campaign (this value can be inferior to the AMOUNT values, since campaigns continue to be active after the funding goal is reached).
- % REACHED - Indicates the percentage of funding as in relation to the minimum funding goal defined by the campaign’s promoters.
- REACH DATE - Indicates the date on which the minimum funding goal was reached.
- DAYS TO REACH - Results of the calculation of the days needed for the campaign to reach its minimum funding.
- NUM REWARDS OFFERED - Indicates the diversity of rewards offered per campaign (ie how many different types of reward were available).
- TOTAL REWARDS CHOSEN - Indicates the total of rewards (of any type) that were selected by backers when contributing with specific amounts to a given campaign.
- TOTAL REWARDS RESIGN - Indicates the total of donations per campaign where users specifically opted not to receive the reward (of any type) in exchange. This can be understood as those donors that wanted to help a given project, without the need to receive something in exchange as campaign rewards.
- AMOUNT RETURNED - Indicates the total money that is returned by Goteo to backers bank accounts after an unsuccessful campaign finishes (ie fails to reach the minimum amount on time).
- AMOUNT REINVESTED - Indicates the total amount of money that is kept in the Goteo accounts of those users opting (when donating) to reinvest the money in other projects where the indicated campaign is unsuccessful.
- AMOUNT BACKED - Regarding campaigns with match-funding modality, indicates the total money donated by individual backers (the same as for those campaigns with no match-funding scheme).
- AMOUNT MATCHED - Indicates the total money that has been added by the “matching” institution of a given match-funding campaign.

### Goteo categories descriptive

This set of tables covers the different categories and criteria for relating campaign social commitments (defined by Goteo users), SDGs numeration (double coded, automatic by Goteo staff and manually afterwards by researchers) and the three footprints (defined by Goteo staff).

- SDG ID - Numeric identifier for each of the 17 Sustainable Development Goals.
- SDG TITLE - Title of each SDG value.
- SDG URL - URL for each SDG description on the UNs website (https://www.un.org/sustainabledevelopment/).
- SC ID - Unique numeric identifier related to the social commitment of the campaign, as selected by the users when describing it via the Goteo form.
- SC TITLE - Title of the given social commitment, as defined by the Goteo user.
- FP ID - Footprint identifier: automatic assignment of values depending on the social commitment category of each campaign.
- FP TITLE - title of the given footprint value, as defined by Goteo staff.

### Goteo campaigns results

This third dataset covers a series of preliminary results derived from combining some of the previous data, in order to observe relevant variables and relationships between modalities of crowdfunding, social commitments and SDGs, among others.

- **Social Commitment clustering**

- PROJECTS (count) - Number of projects under the same cluster of equivalent values (only considering successful campaigns).
- FUNDING TYPE - Regular crowdfunding behaviour is reflected by the value NO MATCHFUNDING + NO MATCHER, and dynamic match-funding (MATCHFUNDING) or with funds added by a matcher institution after the minimum goal was reached (MATCHER).
- SC TITLE - Title of the given social commitment, as defined by the Goteo user.
- UNIQUE DONORS (median) - Value indicates the total number of backers who donated to the campaign (including matching institutions).
- % REACHED (median) - Indicates the percentage of funding as in relation to the minimum funding goal defined by the campaign promoters.
- AMOUNT (median) - Indicates the final amount of money received by the campaigns (in euros).
- DAYS TO REACH (median) - Results of the calculation of the days needed for the campaign to reach its minimum funding.

- **Datafile successful campaigns -** The same fields as “Social Commitment clustering” but prior to grouping campaigns (shows the total of 408 successful campaigns).
- **SDG stats**

- SDG ID - Reflects each SDG after calculating its distribution among the different campaigns (pondering its presence in the total number of projects).
- PROJECTS (count) - Number of projects which have a specific SDG assigned.

- The remaining fields are the same as the “Social Commitment” clustering
- **SDG correlations -** This table calculates the variations between SDGs automatically assigned to each of the campaigns of the dataset and the second round of manual coding.

### Goteo donations detail

This table reflects the details of donations to each campaign: time, amount donated, relation to match-funding mechanisms and date of the transaction. It also reflects whether the receipt of a reward for the donation was declined by the user (a specific feature of Goteo) and the messages of support received with the donation, if any.

- PROJECT ID - Project identification based on keywords (in different languages, depending on the original version).
- D_STATUS - Values referring to the donation being processed or not: ‘Collected’ means the money was processed finally due to the success of the campaign, while ‘Returned’ is assigned when the campaign didn’t reach the minimum funding goal (following an “all-or-nothing” scheme) and money was returned to users. Finally, ‘Returned to wallet’ indicates those reimbursements that (instead of going back to the user’s bank accounts) are kept in their Goteo ones, as indicated by them previously.
- D_AMOUNT - Refers to the money donated (in euros) by the user.
- D_USER - Coded, anonymous identificator of Goteo users for each donation, except for those who selected to donate anonymously (where value is 0).
- D_DATETIME - Date and time value reflecting the precise time of donation.
- D_REWARD RESIGN - Indicates whether or not the donation reward was accepted in exchange.
- D_CALL: If affirmative, the match-funding call in which the campaign is included.
- D_MATCHER - Indicates the matcher user/institution, in campaigns under a match-funding call.
- D_MATCHES - Indicates a match-funding donation which matches a previous one, from a match-funding institution in campaigns under a match-funding call.
- D_MATCHED - Indicates a regular donation that subsequently receives match-funding, in campaigns under a match-funding call.
- D_SUPPORT MSG - Content of messages of support from backers to the campaign they are donating to, if they sent one (in the original language).

### Goteo variable statistics

This additional dataset reflects the different variables of the main dataset regarding Goteo crowdfunding and SDGs, with overall sums or Skewness and Kurtosis statistical analyses to characterise its variability.

## Technical Validation

**Fig. 2:**
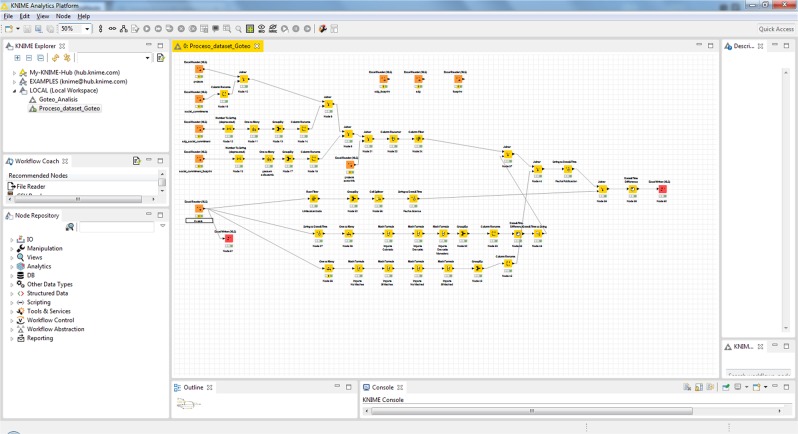
KNIME workspace reflecting the elaboration of the dataset.

**Fig. 3:**
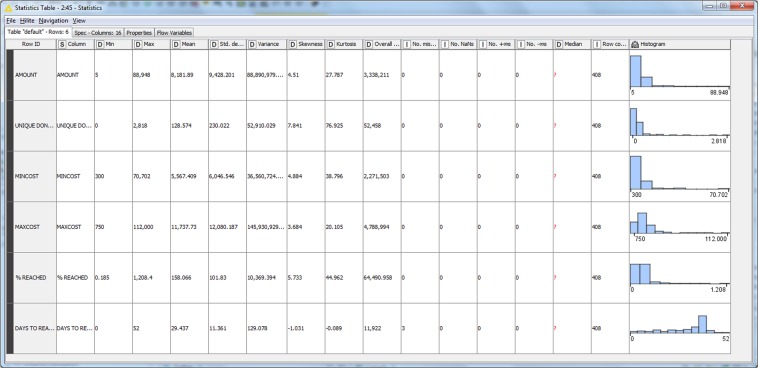
Screenshot of main statistics of the variables related to contributions (KNIME).

In the process of obtaining, coding, combining and preparing the different datasets we performed the following tasks:

1. Extracting information from the Goteo platform information system through API calls (http://developers.goteo.org/doc/) to different tables of the information system:

- Project register

- “projects” table
- “Social_commitments” table
- “Sdg_social_commitment” table
- “Social_commitment_footprint” table

- Register of donations
  - “Invests” Table

(2) Construction of a table containing statistics on the activity of projects registered as of 06/15/2019 (Fig. 2) using the Knime tool (https://www.knime.com/).

(3) English translation of descriptive text fields (previously in the language chosen by the user).
(4) Construction of dichotomous variables based on the possible SDGs that can be assigned to each campaign, depending on the “SOCIAL_COMMITMENT” field (dichotomous variables: sdg2, sdg3, sdg4, sdg5, sdg6, sdg7, sdg8, sdg9, sdg11, sdg12, sdg14, sdg15 and sdg16). Manual typing of campaigns according to the SDGs with which they are related (dichotomous variables: SDG_n for n = 1 to 17).
(5) Estimation of the main descriptions for the quantities related to the amount of contributions recorded in the dataset (Fig. 3).

(6) Estimation of the correlation between SDG variables created from the values registered in “SC ID” and the new SDGs coding variables (n) (Table 1).

**Table 1:** Correlation between SDGs variables according to automatic classification and SDG_N according to the research team.

sdg	sdg_1N	sdg_2N	sdg_3N	sdg_4N	sdg_5N	sdg_6N	sdg_7N	sdg_8N	sdg_9N	sdg_10N	sdg_11N	sdg_12N	sdg_13N	sdg_14N	sdg_15N	sdg_16N	sdg_17N
sdg_2	0.13	0.09	0.25	0.3	−0.13	−0.1	−0.18	0.09	−0.07	0.01	0.06	−0.26	−0.22	−0.13	−0.22	−0.14	−0.02
sdg_3	0.14	0.09	0.24	0.3	−0.12	−0.1	−0.18	0.1	−0.08	0.01	0.06	−0.26	−0.22	−0.13	−0.21	−0.14	−0.02
sdg_4	0.15	0.1	0.16	0.33	−0.1	−0.09	−0.18	0.09	−0.09	0.03	0.1	−0.23	−0.22	−0.14	−0.19	−0.09	−0.01
sdg_5	−0.13	−0.14	−0.32	−0.18	0.21	−0.07	−0.12	0.03	−0.02	0.03	0.16	−0.05	−0.18	−0.14	−0.27	0.23	−0.01
sdg_6	−0.04	−0.04	0.24	−0.09	−0.08	−0.03	0.01	0.02	0.08	−0.03	−0.12	−0.07	0.01	0.05	−0.07	−0.13	−0.02
sdg_7	−0.08	−0.01	−0.09	−0.17	−0.13	0.2	0.37	−0.03	0.03	−0.21	−0.15	0.38	0.48	0.32	0.59	−0.18	0.07
sdg_8	0.15	0.1	0.16	0.33	−0.1	−0.09	−0.18	0.09	−0.09	0.03	0.1	−0.23	−0.22	−0.14	−0.19	−0.09	−0.01
sdg_9	0.15	0.1	0.16	0.33	−0.1	−0.09	−0.18	0.09	−0.09	0.03	0.1	−0.23	−0.22	−0.14	−0.19	−0.09	−0.01
sdg_11	0.09	0.03	−0.05	0.19	0.16	−0.16	−0.34	0.02	−0.06	0.19	0.21	−0.3	−0.44	−0.31	−0.49	0.23	−0.05
sdg_12	−0.08	−0.01	−0.09	−0.17	−0.13	0.2	0.37	−0.03	0.03	−0.21	−0.15	0.38	0.48	0.32	0.59	−0.18	0.07
sdg_14	−0.08	−0.01	−0.09	−0.17	−0.13	0.2	0.37	−0.03	0.03	−0.21	−0.15	0.38	0.48	0.32	0.59	−0.18	0.07
sdg_15	−0.08	−0.01	−0.09	−0.17	−0.13	0.2	0.37	−0.03	0.03	−0.21	−0.15	0.38	0.48	0.32	0.59	−0.18	0.07

(7) Evaluation of the internal coherence of the manual classification of projects (SDG_Ns) by means of the factorial analysis of main components with a rotated solution (Varimax criterion) via the R program (https://www.r-project.org/) (Table 2).

**Table 2:** Correlation between SDG_Ns and Factors in rotated solution (Varimax criteria).

**Factor**	**Sdgs**	**Description**	Correlation between Factors and SDGs									
			**F1**	**F2**	**F3**	**F4**	**F5**	**F6**	**F7**	**F8**	**F9**	**F10**
F1	sdg_17N	Strengthen the means of implementation and revitalize the global partnership for sustainable development	**0.71**	0.15								−0.16
	sdg_14N	Conserve and sustainably use the oceans, seas and marine resources for sustainable development	**0.62**	0.14				−0.17	−0.10		−0.15	0.19
	sdg_6N	Ensure availability and sustainable management of water and sanitation for all	**0.54**			0.12						
F2	sdg_12N	Ensure sustainable consumption and production patterns		**0.62**			−0.16	0.17	−0.14			−0.11
	sdg_15N	Protect, restore and promote sustainable use of terrestrial ecosystems, sustainably manage forests, combat desertification, and halt and reverse land degradation and halt biodiversity loss	0.25	**0.54**	−0.17				−0.17	−0.28	−0.18	0.26
	sdg_7N	Ensure access to affordable, reliable, sustainable and modern energy for all	0.40	**0.45**				−0.13		0.19		
	sdg_13N	Take urgent action to combat climate change and its impacts*	0.35	**0.43**	−0.19		−0.11	−0.28			−0.16	0.26
F3	sdg_10N	Reduce inequality within and among countries		−0.11	**0.97**	0.14						0.14
	sdg_5N	Achieve gender equality and empower all women and girls			0.31				0.17	−0.13	0.17	
F4	sdg_1N	End poverty in all its forms everywhere			0.11	**0.89**						−0.11
	sdg_2N	End hunger, achieve food security and improved nutrition and promote sustainable agriculture				**0.47**			0.11	0.11	0.25	
F5	sdg_3N	Ensure healthy lives and promote well-being for all at all ages		−0.14		0.12	**0.96**		−0.14			
F6	sdg_8N	Promote sustained, inclusive and sustainable economic growth, full and productive employment and decent work for all						**0.64**		0.13	−0.12	
F7	sdg_16N	Promote peaceful and inclusive societies for sustainable development, provide access to justice for all and build effective, accountable and inclusive institutions at all levels		−0.16	0.13		−0.12		**0.66**			
F8	sdg_9N	Build resilient infrastructure, promote inclusive and sustainable industrialization and foster innovation						0.12		**0.62**		
F9	sdg_4N	Ensure inclusive and equitable quality education and promote lifelong learning opportunities for all		−0.13		0.15		−0.11			**0.47**	
F10	sdg_11N	Make cities and human settlements inclusive, safe, resilient and sustainable										**−0.38**
